# Role of Cell Block Technology as an Adjunct to Fine Needle Aspiration in Evaluating as well as Differentiating Liver Lesions

**DOI:** 10.30699/IJP.20201.522897.2569

**Published:** 2021-07-06

**Authors:** Sujata Mallick, Mahasweta Mallik, Rabindra Nath Chatterjee, Puskar Shyam Chowdhury

**Affiliations:** 1 *Department of Pathology, KPC Medical College, West Bengal University of health Sciences, Kolkata, India*; 2 *Department of Pathology, Nalanda Medical College, Assistant professor, Aryabhatta Knowledge University, Patna, India*; 3 *KPC Medical College, West Bengal University of Health Sciences, Kolkata, India*

**Keywords:** Cell block, Cytopathology, Diagnostic utility, Immunohistochemistry

## Abstract

**Background & Objective:**

Liver lesions are difficult to diagnose and to differentiate primary from metastatic carcinoma, while Biopsy has its limitations. Cell block technology is easily accessible with high diagnostic accuracy. Our aim is 1) To find the role of cell block technology as an alternative to biopsy in identifying liver lesions; 2) To find the efficacy of cell block along with immunohistochemistry (IHC) and ancillary studies in differentiating primary from metastatic lesions; 3) To identify the site of origin of metastatic lesions. This is a descriptive study undertaken in two tertiary care hospitals over a period of three years.

**Methods:**

Retrospective review of adequate samples from fine needle aspirations from liver lesions under radiological coverage, converted into cell block was done. IHC was applied as needed. Usefulness of cell block preparation was evaluated, and the final diagnosis correlated with the biopsy results.

**Results:**

Analysis of 323 cases found sensitivity of 98.75% and positive predictive value of 99% for all lesions. Sensitivity for metastatic carcinomas was slightly more than hepatocellular carcinoma. However, accuracy of cell block results for individual metastatic lesions and site of origin was less. IHC and morphological pattern worked as an important adjunct in the final diagnosis. On the other hand, contribution of viral markers as a supplement in the final work up was ambiguous.

**Conclusion:**

High precision of validity results of cell block technology in comparison with biopsy highlights its pivotal role in conjunction with supportive tests for diagnosing and differentiating liver lesions as well as identifying primary sites in liver metastasis.

## Introduction

Carcinoma of liver has a prevalence of 2-8% worldwide (1). Metastatic lesions are more common than primary tumors arising from the liver. Most of the liver masses prototype can be suspected by the clinician with history, signs and symptoms, exami-nation and correlation with radiological aids like USG, CT or MRI. However, confirmation needs a definitive pathological report, previously considered to be a histopathological report following a biopsy (1, 2). Can that be replaced by FNAC with cell block with or without ancillary studies especially in resource limited areas? This is applicable more in cases of liver lesions because of the following reasons: a) Firstly, as com-pared to the other common site for secondaries, that is, lungs, a liver biopsy is technically more difficult and also has more chances of complications including massive bleeding and biliary peritonitis which can lead to death. b) Secondly, primary hepatocellular cancer, specially the poorly differentiated forms is difficult to differentiate from poorly differentiated metastatic carcinomas without the help of markers (immuno-histochemical studies) even in biopsy samples as the morphology appears similar (2). Of the primary cancers of liver, Hepatocellular Carcinoma (HCC) is the most common. HCC develops mostly after chronic hepatitis (Hep B, Hep C infection). FNAC from liver mass can be obtained either blindly or by the aid of imaging technique. FNAC is quick, easy and helps the oncologist to plan out the management of patients. To differentiate between benign and malignant as well as primary and metastatic liver lesion is important because treatment approach varies in these cases, as does prognosis.

Diagnostic sensitivity of FNAC of liver varies from 67-100% and specificity 93-100% (3). So FNAC has gained increased acceptance as surgical procedures are invasive and requires general anesthesia and hospitalization. The yield of FNA sampling in some cases is scanty and may not provide sufficient information for an accurate diagnosis as the histological architecture is lost. Thus, the major drawbacks include the risk of false negative and indeterminate results (4, 5). This leads to diagnostic dilemma especially in differentiating primary and metastatic hepatic tumors and also primary and regenerating liver nodules. FNAC is cost effective, rapid, minimally invasive and yields better architectural pattern and morphological feature with cell block (6). Here, we make an attempt to overcome the deficiencies of FNAC using cell block technology as an adjunct and compare that with a core needle biopsy. This technique refers to processing of sed-iments or grossly visible tissue fragments from cytolo-gyical specimens into paraffin blocks which can be further processed, cut and stained by the same methods used for routine histopathology. Cell block preparation has helped in studying the architecture and also performs immunohistochemistry and special staining, if required (4, 7, 8). If properly done, it is very helpful especially using a small-bore tube and essentially converts cytology to histopathology, thus can be called Fine needle aspiration histopathology. Although FNAC with cell block may be costlier than a biopsy, it is logistically easier on the patients and has a much better compliance as sometimes biopsy has a negative psychological impact. Though final diagnosis in most difficult cases still remains through trucut biopsy, which is a minimally invasive procedure under anesthesia, requiring a TruCut needle of 18 gauze size or an automatic biopsy gun and biopsy material is obtained after an ultra-short incision.

The Aim of this study was to evaluate the scope and accuracy of cell block following FNAC with or without immunohistochemistry along with ancillary studies for diagnosing various liver lesions (especially SOL, space occupying lesions). Also, we aimed at evaluating the role of cell block for differentiating primary hepatic malignancy from metastatic lesions of the liver along with the use of cell block as an adjunct to FNA in sub typing the various metastatic carcinomas and identifying the source or the origin of the malignancy.

## Material and Methods

**Fig. 1:**
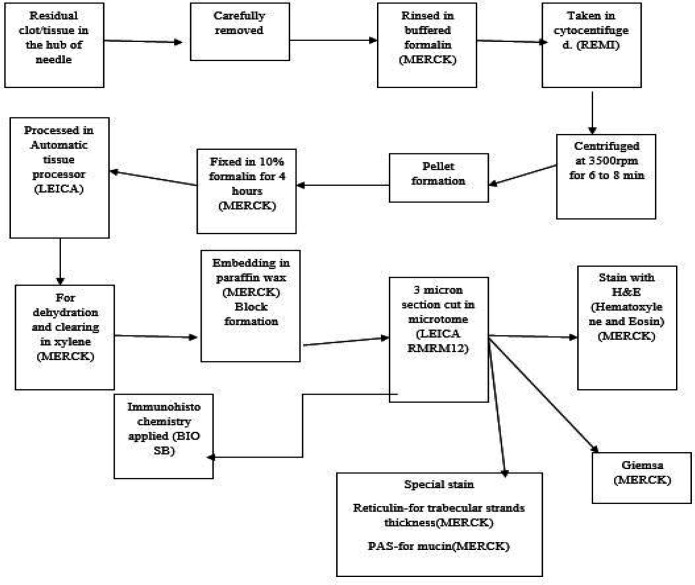
Illustration to show cell block formation methodology in our laboratory

This is a retrospective descriptive study carried out at both KPC medical college, Kolkata and Medical College, Patna over a period of 3 years. As it was a retrospective study no ethical issue or patient consent was needed. A detailed previous history of any other preexisting liver disease and record of serological viral marker, where available, were collected from the surgery department. FNAC was carried out either blindly or with USG/CT guidance in the radiology department. Direct air-dried smear were stained with MGG. Some smears were immediately fixed in 95% alcohol and stained with Pap. The remaining material in the syringe was allowed to clot to form cell block, where aspiration was adequate for cell block formation (Figure 1).

Results were analyzed by two independent senior pathologists and a final conclusion of the diagnosis was derived after discussions with a third senior faculty. 

All the procedures were performed following the standard operating procedures with routine and con-sistent checks to identify and address various types of errors and omissions, ensuring data integrity, correct-ness and completeness of all the available records. The quality control checks included accurate patient identification, proper fixation time, adequate processing measures, appropriate embedding techniques, precision in microtome sectioning, unacceptable artifacts and regular inspection of controls used in IHC and special stains to determine the correctness in our method.

Statistical analysis was done using Chi-square to compare various parameters. The P-value was calcula-ted using the sampling distribution of the test statistics under the null hypothesis and our sample data as in a two-sided test. In our analysis, an alpha of 0.05 was used as the cut off for significance. When the P-value was less than 0.05, we rejected the null hypothesis that there is no difference between the means; thus, we concluded that a significant difference exists. So, in our study, P-value below 0.05 was taken as significant and over 0.05 as not significant. Fischer’s exact test was also done to compare various parameters in the patients.

## Results

Out of 416 cases who underwent guided FNAC from liver, 15 cases were considered inconclusive for reporting due to very scanty cellularity or blood only aspirate. Among the adequate aspirations which were 401 in number, the aspirate was enough to make cell block in 349 cases. Others were reported on FNAC as benign or malignant and were not included in our study.

Age range varied from 42 to 84 years, with a mean age of 65.5 years. Hepatocellular carcinoma was in the range of 48-84 years with a mean of 67.2 years while metastatic age range was 42-81 years with a mean of 58.4 years. Highest amount of inadequate and inconclusive smears was when the lesion size was <1 cm. Male to Female ratio was 6:4.

**Fig. 2:**
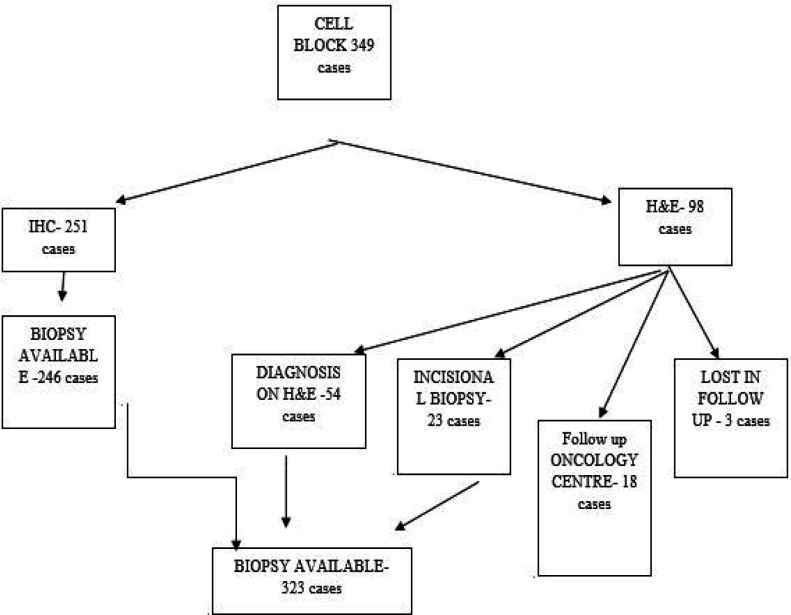
Distribution of the cases in study population with selection of sample population

 In 251 out of 349 cases, immunohistochemical study could be done on cell block preparation. Among the rest 98 cases, 54 did not require IHC due to clear morphology on H&E staining for a final diagnosis. Of these cases, 23 were non-compliant for IHC study, mostly due to economic reasons and decided to go for direct incision biopsy as it is the gold standard. Of the patients, 18 opted for further investigation and treatment in an oncology center, while 3 were lost in follow up after H&E reporting on cell block. So, the total biopsy results were obtained for 323 cases which remained our study sample (Figure 2). 

**Fig. 3:**
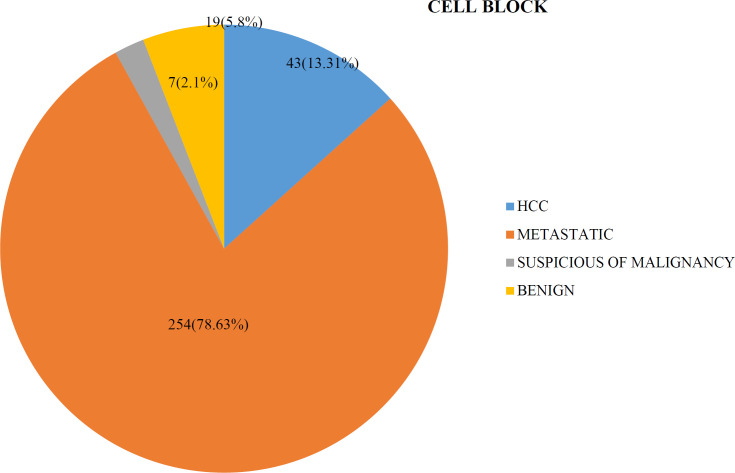
Distribution of the cases in cell block preparation

On cell block, with or without immunohistoche-mistry, 43 cases (13.31%) were positive for hepato-cellular carcinoma, 254 cases (78.63%) were positive for metastatic lesions, 7 cases (2.1%) were suspicious of malignancy and 19 cases (5.8%) were designated as benign lesions (Figure 3).

**Fig. 4:**
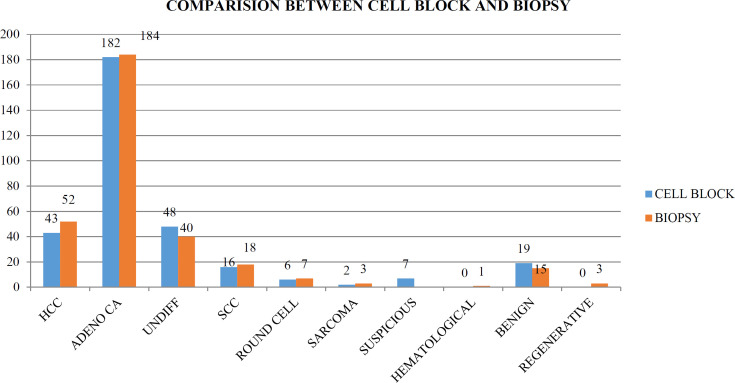
Comparison of the cell block results with the biopsy results

Individual comparison of cell block results with that of biopsy, which is the final diagnostic tool, showed a few discrepancies in interpretation of individual lesions. In biopsy, 52 cases (16.09%) were primary hepato-cellular carcinoma, 253 cases (78.32%) were metastatic lesions while 15 cases (4.64%) were actually benign and 3 cases (0.9%) were regenerative nodules (Figure 4). A detailed correlation of individual lesions is given in the Table 1. 

**Table 1:** Correlation of the cases in cell block with that of biopsy with immunohistochemical markers, control used and source of origin of metastasis

Cell block					Biopsy		
HCC (43) Control-known HCC case Marker-Hep Par 1, pCEA, α feto protein	Poorly differentiated	1			HCC		1
	others	42			HCC		42
METASTATIC (254)	a) Adenocarcinoma Control-Appendix Marker-CK7, CK20,pCEA	Poorly differentiated		13	Adenocarcinoma Gall Bladder-3		4
					Adenocarcinoma others- Colon-6 Stomach-1 Ovary-1 Pancreas-2 HCC-0		7 4 1 0 2 2
					HCC		2
		Well -mod differentiated		169	Adenocarcinoma others Colon-58 Stomach-24 Pancreas-10 Ovary-0 Unknown Primary-2		94 56 23 12 1 2
					Adenocarcinoma Gall Bladder-75		75
	b) undifferentiated Control- known poorly differentiated carcinoma Marker-CK7,CK20,pCEA,αfeto protein,SMA,HepPar1	48			Undifferentiated-42		40
					HCC-2		3
					Adenocarcinoma others-0		2
					SCC-2		2
					Sarcoma-3		1
	c)SCC Control-Seborrheic keratosis Marker-CK7,CK20	16			SCC-16		16
	d)Round cell Control-Ewings sarcoma Marker-Synaptophysin, Chromogranin	6			Round cell-6		6
	e) sarcoma Control-Fibroid Marker-SMA	2			Sarcoma-1		2
Suspicious of malignancy Control-All IHC-All	7				HCC	4	
					Regenerative nodule	3	
Benign (19)	Inflammatory		5		Round cell/Neuroendocrine tumor	1	
					Hematological malignancy	1	
					abscess	3	
	Necrosis		4		Adenocarcinoma others	1	
					Adenocarcinoma GB	1	
					Abscess	2	
	benign		10		cirrhosis	6	
					abscess	4	

There was occasional variance between both the results of cell block and biopsy in almost all lesions, however the disparity was obvious in undifferentiated carcinoma with eight false positive cases. Hepatocellular carcinoma was diagnosed when polygonal cells with eosinophilic cytoplasm, large vesicular nucleus with prominent nucleoli were seen in the smears. When smears showed malignant cells arranged in loose clusters or sheets of pleomorphic cells with moderate to abundant cytoplasm, they were diagnosed as metastatic adenocarcinoma. Adenocarcinoma metastasis from GIT, ovary, and pancreas with metastatic adenocarcinoma from gall bladder was differentiated with IHC and other ancillary studies like radiological imaging, history along with clinical examination of the patient. Similarly, for undifferentiated metastatic carcinoma, the site of origin of primary focus was determined by considering all the above parameters. Round cell tumor had tight clusters of monomorphic cells with nuclear molding and scanty cytoplasm. Sarcoma metastasis showed oval to spindle cells with indistinct cytoplasm. Regenerative nodules had hyperplastic hepatocytes with no distinctive cyto-architectural features and were mistaken as suspicious for malignancy on cytology. Hematological diagnosis was also missed in cell block technique. Due to aspiration from necrotic area, a few cases of metastatic adenocarcinoma were missed. Immunohistochemistry was utilized to arrive at the final diagnosis, as and when essential. 

Morphology was observed from the smears obtained with MGG, PAP and H&E routinely from the cell block preparation. Special stain was PAS (to look for mucin) and reticulin (to look for trabecular strand) was also performed on cell block preparation. Table 2 and 3 were utilized to differentiate between hepatocellular carcinoma, poorly differentiated metastatic carcinoma, moderately to well differentiated metastatic carcinoma and benign lesions of the liver.

**Table 2:** Differentiation of the tumors based on morphology

Morphology	HCC	Poorly differentiated Metastatic carcinoma	Moderately differentiated to well differentiated metastatic carcinoma	Benign lesion
1) Cytological pattern				
Trabecular pattern	++	+	-	+-
Hepatocytic appearance	+	+-	-	++
Intracellular bridge	+	+-	-	+-
2) Gland formation (in cell block /cytology)	+/-	+/-	+++	-
3) Special stains				
Reticulin stain	++	+-	-	+++/-
P & E	-	+/-	+-++	-

**Table 3:** IHC study on the liver carcinomas

	Hepatocellular carcinoma	Poorly differentiated metastatic Carcinoma	Moderately differentiated to well Differentiated metastatic carcinoma	Round Cell/ Neuroendocrine tumor	Sarcoma	Benign lesion
CK7	-	+	++	-	-	-
CK20	-	+-	+	-	-	-
Hep Par-1	+	-	-	+-	-	+-
pCEA	+-	+-	+	-	-	-
α feto protein	++	+-	-	-	-	+-
Synaptophysin	--	+-	+-	+	-	-
Chromogranin	--	+-	+-	++	-	-
SMA	-	-	-	-	+	-

In morphology, 

1) Cytological features used were a) trabecular pattern (*P*=0.0001) (b) hepatocytic appearance (large polyhedral cell with abundant cytoplasm and nuclear character) (*P*=0.0000) C) Intracellular bile (*P*=0.005);

2) Gland formation-well-formed cluster of glands mostly seen in moderately to well differentiated adenocarcinoma.

3) Special stain- 

a) Reticulin stain was used to see the trabecular strands thickness which was usually present in hepatocellular carcinoma and some benign lesions whereas it was absent in metastatic carcinoma.

b) PAS to look for mucin was present in some varieties of moderately differentiated to well differentiated metastatic carcinoma (mucin secreting adenocarcinoma) whereas it was universally absent in hepatocellular carcinoma and benign lesions.

Immunohistochemistry was done with CK7 & CK20, Hep Par-1 and p CEA staining. All cases of hepato-cellular carcinoma were positive to Hep Par-1 and negative for CK7 and CK20. pCEA was equivocal. All cases of moderately to well differentiates adenocarci-nomas were strongly positive for CK7 and weakly positive for CK20 and pCEA. Hep Par-1 was uniformly negative. Poorly differentiated metastatic carcinoma was positive for CK7 and negative for Hep Par-1. CK20 and pCEA were equivocal and were not helpful. Benign lesions of the liver were Hep Par-1 positive except for the abscess (2 cases). α feto protein was highly positive for hepatocellular carcinoma but poorly differentiated lesions also showed focal positivity in certain cases. Neuroendocrine markers were positive for round cell tumors with chromogranin displaying stronger positivity than synaptophysin. A few equivocal results were also discerned in metastatic lesions. SMA was positive in sarcomatous lesions which along with morphology helped in diagnosis. All the other markers were negative.

Serological studies of viral markers were documented from the patient’s history recorded in the surgical department and were available for 16 cases of metastatic carcinoma and 39 cases of HCC. Serum viral markers including HbsAg (Australian antigen) and anti HCV antibody checked. Viral assay for both Hepatitis B (titer of Hep B DNA) and Hepatitis C (titer of Hep C RNA) were done. While either hepatitis B or Hepatitis C were present, in cases of hepatocellular carcinoma (39/323) consistently more often than in both poorly differentiated and moderately to well differentiated metastatic adenocarcinoma, it is not helpful to differentiate between benign liver disease and hepatocellular carcinoma. The number (16/323) of viral markers done in the metastatic group was very less, however an increased percentage was found to be positive in those tested as it was done only in cases showing liver damage (obtained by history and elevated liver enzymes) (Table 4).

A detailed statistical analysis showed sensitivity of all the lesions diagnosed through cell block method to be 98.75% with positive predictive value of 99% and P-value highly significant at <0.00001. Diagnosing metastatic carcinoma was also very accurate with positive predictive value of 99.2%. Primary lesion like hepatocellular carcinoma with 100% positive predictive value, 91.5% sensitivity and significant P-value had very precise results on cell block. However, differentiating the various types of metastatic lesions on cell block was less on target with accuracy ranging from 66.66% to 100% for various carcinomas (Table 5). 

**Table 4:** Viral marker correlation with the liver cancer

	Hepatocellular carcinoma	Poorly differentiated metastatic Carcinoma	Moderately differentiated to well Differentiated metastatic carcinoma	Benign lesion
HbsAg	+/-	+/-	+/-	+/-
HepB DNA	+/-	-	-	+/-
Anti HCV Ab	+/-	+/-	+/-	+/-
HepC RNA assay	+/-	-	-	+/-

**Table 5:** Statistical analysis of the cell block and biopsy

	Analysis of all lesion in cell block with biopsy	Analysis of hepatocellular carcinoma in cell block with biopsy	Analysis of metastatic carcinoma in cell block with biopsy	Analysis of different types of metastatic lesion in cell block with biopsy	
Sensitivity	98.75%	91.5%	98.44%	Accuracy of Metastatic Adenocarcinoma	98.9% *P*<0.00001
				Accuracy of undifferentiated CA	100% *P*<0.00001
Specificity	83.33%	100%	97.0%	Accuracy of SCC	88.88% *P*<.00001
Positive predictive value	99.0%	100%	99.2%	Accuracy of round cell carcinoma	85.7% *P*<0.00001
P-value	<0.00001	<.00001	0.00001	Accuracy of sarcoma	66.66% *P*<0.00672

## Discussion

FNAC from liver has proven to be a better diagnostic tool than core needle biopsy or open biopsy in terms of cost, procedure and early diagnosis (9). Liver abnormalities are first confirmed by palpation, USG or CT scan and then proceeded for FNAC.

Tumor size (benign or malignant hepatic lesion) bigger than 5 cm had better successful aspiration and greater accuracy than tumor <1 cm. Similar results depending on tumor size is detected by Voit* et al. *and Willems* et al. *(10, 11). For proper diagnosis FNA from liver lesion and their cell block preparation has to have proper cellularity. According to a study by Sukumaran* et al. *(5), 438 out of 638 cases were adequate for cell block whereas 69 were inadequate and 131 inconclusive. Their age range was from 0-88 years. Whereas our study showed 349 out of 401 cases as adequate for cell block preparation and our age range was 42-84 years with a mean age of 65.5 years. The study by Mathew* et al. *(4) showed age range from 25-78 years with mean age at 58.5 years. The imaging results of most of the cases, comprising both hepatocellular carcinoma and metastatic lesion was a solitary SOL. In our study 34 cases out of 43 HCC (79%) cases presented as solitary SOL and 219 out of 254 (86.2%) metastatic carcinoma presented as solitary SOL. According to the study by Mohmmed* et al. *(1) 66% of HCC cases and 65.5% of metastatic cases presented as solitary SOL.

The earlier the diagnosis of HCC, the better is its prognostic implications. So, differentiating primary HCC from metastatic carcinoma helped in facilitating early treatment modalities. The results of Mohmmed* et al. *(1) showed 39% of cases as malignant, 27.9% as bloody sample and 3.8% as normal hepatocytes. Among malignant cases, 25.7% were hepatocellular carcinoma, 42% were metastatic adenocarcinoma, 2.9% were spindle cell sarcoma and 1% hepato-blastoma. Sukumaran* et al. *(5) showed adeno-carcinoma to be the most common metastatic tumor at 83% followed by neuroendocrine tumor (15 cases), then poorly differentiated carcinoma with 1 or 2 cases each of other tumor like GIST, neuroblastoma, SCC and sarcomas. Our study follows the same trend of primary and metastatic carcinoma with mild variations in the unusual tumors’ presentations. Cell block provides information like trabecular sinusoidal pattern, pseudo acini, arteries and absent reticulin framework which is adequate for differentiating well differentiated HCC from regenerating hepatocytes and also for differentiating poorly differentiated HCC from poorly differentiated metastatic carcinoma. Cytological features of HCC according to Sukumaran* et al. *(5) are the three primary criteria like a high N/C ratio, a trabecular arrangement pattern, and atypical naked nuclei.

Metastatic cases in our study were the highest (78.6%) similar to Tao* et al. *(12) whose study of 1037 cases showed 75% metastasis. In the present study, no recorded complications were present following FNAC, however, some authors have reported fatal bleeding in chronic liver disease, needle tract seedling and biliary venous fistula (13, 14). Intrahepatic hematoma was reported by Lundquist (15).

Immunohistochemistry helps in classification and prognostication of hepatocellular tumors which is shown in the study by Cheuk-lam Lo* et al. *(16). Careful histologic observations and judicious use of IHC acts as a useful adjunct in the right diagnosis of hepatic masses, highlighted in the study by Walther* et al. *(17). CK7 and CK20 plays an important role in the diagnosis of metastatic carcinoma of unknown primary site. It provides diagnostic guidance in approximately 90% of undifferentiated malignant tumor though morphology also plays a fastidious role according to the study by Selves* et al. *and Fan* et al. *(18, 19). They found commercially available Hep Par1 antibody to be a sensitive marker for HCC in paraffin embedded sections on 676 tumors including 19 cases of HCC out of which 18 were positive for HepPar 1. Studies by Grazi* et al. *and Edoo* et al. *(20, 21) found sensitive serum markers.

pCEA is a useful contributor to the diagnosis of small liver tumor still amenable by surgery. Wang* et al. *and Nguyen* et al. * (22, 23) showed the importance of Hep Par1 and pCEA for distinguishing hepatocellular carcinoma vs metastatic adeno-carcinoma in liver fine needle aspirates. Our study showed all cases of Hepatocellular carcinoma to be positive for Hep Par 1 and negative for CK7 and CK20. pCEA was equivocal. Metastatic carcinomas were strongly positive for CK7 and weakly positive for CK20 and pCEA with Hep-Par negative. Bialecki* et al. *(24) stated that serum AFP levels can be helpful, if markedly elevated in the surveillance of high-risk individuals for HCC. Behne* et al. *and Murugavel* et al. *(25, 26) correlated the same. Colquhoun* et al. *(27) and Veenendaal* et al. *(28) found chromogranin A to be 100% specific and a highly sensitive marker for NETs. Synaptophysin also plays an important role. Hamai* et al. *(29) showed the role of SMA in diagnosing leiomyosarcoma of colon with liver metastasis. Our study found these markers to be very helpful in diagnosing primary HCC and metastatic lesions and supplementary in differentiating metastatic lesions.

 Noh* et al. *(30) found out in their study the relation between chronic HBV and HBC with the development of HCC. Zamor* et al. *(31) believed HBV and HBC led to hepatic fibrosis which further developed into HCC. Their study showed 50% of cases were related to chronic hepatitis with majority residing in Asia. Other studies by Perz *et al.*, Di Bisceglie* et al. *and Yuen* et al. *(32-35) also related the development of HCC due to increased viral load of HBV or HCV. Mendy* et al. *(36) found out that even low-level viremia (200-10000 copies/mL) conferred a significant risk of HCC. Our study showed a positive correlation between increased viral load of HBV and HCV with the development of hepatocellular carcinoma, however with a few cases of increased viral load present for even metastatic lesions, a scope for ambiguity remains (although this may be explained by the increased prevalence of chronic hepatitis in our country).

Mathew* et al. *(4) discovered in their study that cell block from FNAC in liver has 71.11% sensitivity, 100% specificity and 71.7% accuracy. Other studies showed that sensitivity for diagnosis of hepatic malignancy by cell block is from 75.34% to 93% (32, 35). Our study showed the sensitivity to be 98.75%. No false positive case was present in the study by Homesh *et al.* and Iyer *et al.* (37, 38) though we had 3 false positive cases. Some false negative cases were attributed to repeated aspiration of necrotic material leading to diagnosis of abscess where underlying carcinoma was missed. Various studies have reported specificity by cell block method to be from 69% to 100% (38-40). Our study demonstrated a specificity at 83.3%. Sometimes, differentiating poorly differen-tiated hepatocellular carcinoma and metastatic adenocarcinoma were difficult (41). In a few cases, disorganized hepatocytes and cholestasis from liver parenchyma leads to a cirrhotic picture instead of metastatic carcinoma. Our study had 4 false negative cases with 2 cases appearing as abscess on cell block while other 2 displayed only inflammatory cells. Mohmmed* et al. *(1) found positive predictive value to be 78.8% and negative predictive value at 0% while our study had high range of positive predictive value at 99% which supports the efficacy of this diagnostic method.

Cell block converts a suspicious report into a definitive diagnosis. We have to ask ourselves, “Do we really need to do core biopsy?” Because in resource limited areas cell block is a poor man’s core needle biopsy and can be used as an adjunct to histopathology.

In cell block, architecture of tumor is maintained at places whereas core biopsy can have crush artifact. Even in higher centers, in certain cases, cell block is better than core biopsy, which is formalin fixed, as studies show that formalin can hinder in DNA extraction, especially in molecular studies. However, in pediatric age group, FNAC with cell block can be used in certain cases though core biopsy remains the gold standard in most pediatric tumors. Some believe that biopsy tract seedling using unsheathed needle is probably more common than fine needle aspiration spilling, though there is no proven data. 

## Conclusion

A satisfactory FNAC sample with cell block is a very useful diagnostic tool for evaluation of various liver lesions with high degree of diagnostic accuracy. Also, it reduces the timing, the economic burden and morbidity of the patient.

In cases where diagnosis by FNAC is equivocal, it is recommended to perform FNA with cell block preparation and IHC studies as a part of routine laboratory practice to improve diagnostic precision. Because of its high sensitivity, Cell Block technique is a useful adjunct to routine FNA smear because multiple sections can be cut from a cell block and IHC and special stains can be applied. Viral markers, if available, can be correlated to arrive at the final diagnosis. The combination of cell block with all these adjunct techniques is of immense help in identifying primary carcinoma and differentiating it from metastatic deposits in the liver without any invasive procedure. The source of the primary site in metastatic deposits can be detected which can guide the treatment protocol and even helps in predicting the prognosis.
